# Influencing factors of kinesiophobia in patients after anterior cruciate ligament reconstruction: A scoping review

**DOI:** 10.1097/MD.0000000000045138

**Published:** 2025-10-10

**Authors:** Xuemei Zheng, Mengjing Chang, Wenling Tian, Xiangyue Liu, Dongfa Liao, Huiyun Yuan, Lin Cui

**Affiliations:** a College of Nursing, North Sichuan Medical College, Nanchong, China; b Department of Orthopaedics, The General Hospital of the Western Theater Command of the People’s Liberation Army, Sichuan, China; c College of Nursing, Chengdu Medical College, Chengdu, China; d Department of Nursing, The General Hospital of the Western Theater Command of the People’s Liberation Army, Chengdu, China.

**Keywords:** anterior cruciate ligament reconstruction, influencing factors, kinesiophobia, scoping review

## Abstract

**Background:**

To understand the current status and influencing factors of kinesiophobia in patients after anterior cruciate ligament reconstruction (ACLR), and to provide references for the refined management of kinesiophobia patients by medical workers.

**Methods:**

Guided by the scoping methodology, the Web of Science, Cochrane Library, PubMed, Embase, EBSCO, China National Knowledge Infrastructure, China Science and Technology Journal Database, Wanfang, and China Biomedical Literature were searched from database inception until May 31, 2025. The included literature was integrated and analyzed.

**Results:**

A total of 16 articles were included in the study, involving 1725 patients. The incidence of kinesiophobia in patients after ACLR was relatively high, ranging from 33.33% to 92%. The influencing factors could be summarized into 3 themes, including general condition factors (gender, age, body mass index, family income, employment status, education level, and sleep quality), disease characteristics and treatment factors (postoperative time, timing of surgery after injury, number of injuries, function of the injured knee, pain intensity, muscle activity, and biomechanical indicators), and social psychological factors (pain catastrophizing, psychological state, and self-perceived or self-efficacy scores).

**Conclusion:**

Kinesiophobia is highly prevalent among patients after ACLR, with complex influencing factors. Assessment tools vary between domestic and international studies, and current research remains limited. Future studies should explore the causal relationships between kinesiophobia and its influencing factors, and develop targeted interventions to reduce its incidence, enabling patients to return to sports and society prompt.

## 1. Introduction

Anterior cruciate ligament (ACL) injury is one of the most common knee injuries.^[1]^ According to statistics, more than 400,000 people worldwide need treatment for ACL injury every year.^[2]^ In recent years, with the development of national fitness activities, the incidence of ACL injury has gradually increased.^[3]^ Currently, anterior cruciate ligament reconstruction (ACLR) is the main treatment for restoring knee stability after ACL injury.^[4]^ Studies have shown that 90% of patients who undergo ACLR can recover knee function after surgery, but only 65% of patients can return to their pre-injury level of movement.^[5]^ Among them, sports phobia is considered to be a key factor that hinders and limits patients from returning to sports.^[6]^ Since ACL injuries are mostly sudden acute sports injuries, patients are prone to the stress psychological reaction of “fear of reinjury,” so ACLR patients often develop sports phobia due to concerns about ligament reinjury or poor prognosis. In 1983, Lethem^[7]^ proposed the “fear-exercise-avoidance” model, and Kori^[8]^ 1st defined “kinesiophobia,” which means that after suffering from painful stimulation, individuals are afraid of secondary pain caused by activities, and then develop excessive and irrational fear and behavior towards exercise. Kinesiophobia will directly affect patients’ compliance with exercise rehabilitation, promote muscle atrophy, reduce cardiovascular health, and increase the economic burden on families and society.^[9,10]^ In recent years, although domestic and foreign scholars have actively carried out research on the influencing factors of kinesiophobia in ACLR patients and have achieved certain results, unfortunately, there is still a lack of a comprehensive review of these studies. Due to the relatively limited number of existing literature on kinesiophobia in ACLR patients, its importance has not yet received sufficient attention and attention from medical workers. This article systematically reviews domestic and international studies on the influencing factors of kinesiophobia in patients after ACLR, aiming to promote the advancement of research on post-ACLR kinesiophobia, provide references for subsequent clinical prevention and intervention work, and thereby reduce the occurrence of kinesiophobia.

## 2. Materials and methods

### 2.1. Determine the research questions

Based on the scoping review framework proposed by Arksey,^[11]^ this study systematically analyzed the literature on kinesiophobia in patients after ACLR surgery and summarized the incidence, influencing factors, and assessment tools of kinesiophobia in patients after ACLR surgery. Specific research questions include: What is the current status of kinesiophobia in patients after ACLR surgery? What are the influencing factors of kinesiophobia in patients after ACLR surgery?

### 2.2. Literature inclusion and exclusion criteria

Inclusion criteria were determined according to the PCC principle.^[12]^ Inclusion criteria: research subjects (participants, P) were patients with kinesiophobia after ACLR surgery; concept (C) was an article that investigated the current status and influencing factors of kinesiophobia in patients after ACLR surgery; and context (C) was original research from around the world, with kinesiophobia as the primary outcome measure. Exclusion criteria: studies for which the full text was not available; literature not in Chinese or English; literature that was duplicated or of low quality; and reviews and conference abstracts.

### 2.3. Search strategy

**Figure 1.**
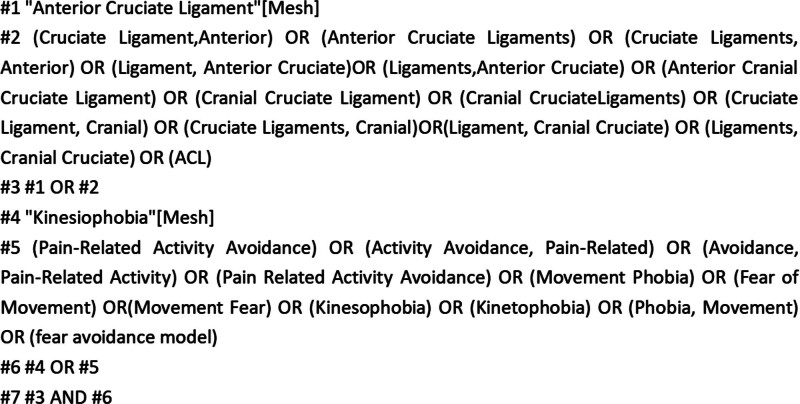
Literature search strategy. ACL = anterior cruciate ligament.

The databases that were searched included PubMed, Web of Science, Cochrane Library, Embase, EBSCO, China Biomedical Literature, China National Knowledge Infrastructure, China Science and Technology Journal Database, Wanfang. The search time is from the establishment of the database to May 31, 2025. The search used a combination of subject headings, free words, and Boolean logic operators. The search keywords were: “kinesiophobia,” “akinesiophobia,” “fear of movement,” “psychophobia,” “fear,” “fear of exercise,” “cruciate ligament,” “knee joint,” “ACL,” “ACLR,” “joint,” and “ligament.” The search strategy is shown in Figure 1 and File S1, Supplemental Digital Content, https://links.lww.com/MD/Q276.

### 2.4. Literature screening and data extraction

Two researchers who had received evidence-based training independently screened the literature and cross-checked it in strict accordance with the inclusion and exclusion criteria. If there was any dispute, a 3rd party could be requested to make a ruling.^[13]^ After the literature was identified, relevant data were extracted, including the following: general characteristics of the literature: author, year of publication. General characteristics of the study: study location, study type, sample size. Research variables and the influencing factors obtained. Evaluation tools. Since this study is a scoping review that only involves systematic collection, screening, and analysis of existing published literature, without involving direct recruitment of human subjects, collection of original clinical data, or intervention in the physical or psychological status of patients, it does not meet the conditions requiring ethical review by an ethics committee or institutional review board. Therefore, ethical approval for this study is waived, and the issue of patient informed consent is not applicable.

## 3. Results

### 3.1. Literature screening process and results

A total of 1199 articles were obtained by searching various databases. After removing duplicates and reading abstracts and titles, 48 articles were obtained, and 16 articles were finally screened after reading the full text.^[14–29]^.

### 3.2. Basic characteristics of included literature

This study focused on kinesiophobia after ACLR and conducted a comprehensive search of major databases, and finally included 16 literature, including 13 cross-sectional studies, 1 case-control study, and 2 cohort studies, with a total sample size of 1725 cases. A brief overview of the included literature is shown in Table 1.

**Table 1:** Basic information of the included literature.

References	Country	Type of study	Number of cases	Evaluation Tools	Influencing factors
Zhao et al^[14]^	China	Cross-sectional study	255	Chinese version of TSK-17	①②③④
Li et al^[15]^	China	Cross-sectional study	240	Chinese version of TSK-17	③⑤⑥⑯
Chen et al^[16]^	China	Cross-sectional study	216	Chinese version of TSK-17	②⑥⑦⑧⑨⑩
He et al^[17]^	China	Case-control study	19	Chinese version of TSK-17	⑯
Li et al^[18]^	China	Cross-sectional study	203	Chinese version of TSK-17	②④⑦⑫
Tajdini et al^[19]^	Sweden	Cross-sectional study	28	TSK-11	⑭⑮
Ohji et al^[20]^	Japan	Cross-sectional study	31	TSK-11	⑮⑯
Rips et al^[21]^	Sweden	Cross-sectional study	140	TSK-17	①⑫
Hartigan et al^[22]^	USA	Cohort study	111	TSK-11	⑪
Baez et al^[23]^	USA	Cross-sectional study	36	TSK-11	⑭
Raizah et al^[24]^	Saudi Arabia	Cross-sectional study	130	TSK-17	①⑬
Knapik et al^[25]^	USA	Cohort study	25	TSK-11	⑭⑰
Chmielewski et al^[26]^	USA	Cross-sectional study	97	TSK-11	⑪⑰
Dudley et al^[27]^	USA	Cross-sectional study	15	TSK-11	⑭
Norte et al^[28]^	USA	Cross-sectional study	77	TSK-17	⑪⑮
Theunissen et al^[29]^	Netherlands	Cross-sectional study	102	TSK-17	①⑤⑫⑯

① : Timing of surgery after injury; ② : self-efficacy or perception score; ③ : psychological state; ④ : pain catastrophizing; ⑤ : body mass index; ⑥ : sleep; ⑦ : education level; ⑧ : non-employed; ⑨ : monthly family income < 3000 yuan; ⑩ : injury ≥ 3 times; ⑪ : injured knee function; ⑫ : gender; ⑬ : age; ⑭ : biomechanical index; ⑮ : muscle activity; ⑯ : pain level; ⑰ : postoperative time.

### 3.3. Assessment tool for kinesiophobia after ACLR

The scale for assessing kinesiophobia in patients after ACLR is derived from the Tampa Scale of Kinesiophobia (TSK) developed by American scholars. The scale focuses on the patient’s cognition and feelings about exercise, physical pain, and injury risk. It contains 17 items, all of which are scored using the Likert 4-point rating method, with scores ranging from 1 to 4 from “strongly disagree” to “strongly agree.” Items 4, 8, 12, and 16 are reverse scored, with a total score of 17 to 68 points. The higher the score, the higher the patient’s kinesiophobia. The criterion for the presence of kinesiophobia is a total score of ≥ 37 points. In the included literature, Rips^[21,24,28,29]^ all used this version of the scale. In 2012, Chinese scholar Hu Wen^[30]^ translated the TSK-17 scale into Chinese. The number of items did not change. The translated scale was tested for reliability and validity. The Cronbach α coefficient of the scale was 0.740 and the test-retest reliability was 0.860. Chinese scholars Zhao Fang^[14–18]^ used the translated TSK-17 scale to assess kinesiophobia. In addition to TSK-17, there are also versions of kinesiophobia scales such as TSK-13 and TSK-11. In 2005, Woby^[31]^ found that some items had low total correlation and the response trend deviated from the normal distribution. Therefore, they designed TSK-11 and compared the psychometric properties with the original TSK scale, proving that the simplified version of TSK-11 is reliable and valid. The scale mainly involves 2 dimensions: avoiding activities and focusing on pain, with a total of 11 items. All items are scored using the Likert 4-level scoring method, with a total score of 11 to 44 points. The criterion for determining the presence of kinesiophobia is a total score ≥ 26 points.

### 3.4. Factors affecting kinesiophobia in patients after ACLR surgery

#### 3.4.1. General influencing factors

Among the included literature, 6 articles^[14,15,18,20,23,28]^ mentioned that gender, age, body mass index, family income, employment status, education level, and sleep quality were influencing factors for the occurrence of kinesiophobia in patients after ACLR surgery, among which gender and education level had a higher frequency. It is shown in Table 2.

**Table 2:** Frequency table of influencing factors.

Category	Influencing factors	Frequency	Included studies
General influencing factors	Gender	3	^[18,20,28]^
	Age	1	^[23]^
	Body mass index	2	^[14,28]^
	Household income	1	^[15]^
	Employment status	1	^[15]^
	Education level	2	^[15,18]^
	Sleep quality	2	^[14,15]^
Disease characteristics and treatment factors	Postoperative time	2	^[24,25]^
	Timing of surgery after injury	4	^[14,21,24,29]^
	Number of injuries	1	^[15]^
	Injured knee function	3	^[21,25,27]^
	Pain level	5	^[14,16,18,19,28]^
	Muscle activity	3	^[17,18,27]^
	Biomechanical indicators	4	^[17,22,24,26]^
Psychological factors	Pain catastrophizing	2	^[14,18]^
	Mental state	2	^[14,15]^
	Self-efficacy rating	2	^[16,18]^
	Self-perceived burden score	1	^[14]^

#### 3.4.2. Disease characteristics and treatment factors

Among the included literature, 14 articles^[14–19,21,22,24–29]^ mentioned that postoperative time, timing of surgery after injury, number of injuries, injured knee function, pain level, muscle activity, and biomechanical indicators were influencing factors for the occurrence of kinesiophobia in patients after ACLR surgery. Among them, pain level and timing of surgery after injury had a higher frequency. It is shown in Table 2.

#### 3.4.3. Social and psychological factors

Among the included literature, 4 articles^[14–16,18]^ mentioned that pain catastrophizing, psychological state, self-perceived burden score, and self-efficacy score were influencing factors for the occurrence of kinesiophobia in patients after ACLR surgery, among which pain catastrophizing had a higher frequency. It is shown in Table 2.

## 4. Discussions

### 4.1. The prevalence of kinesiophobia after ACLR surgery is high, and prevention and timely intervention are needed in advance

The incidence of kinesiophobia in patients after ACLR is high, ranging from 33.33% to 92%. The incidence of kinesiophobia in day surgery patients is 92%, which is much higher than the 33.33% incidence in hospitalized patients. The reason for this difference may be related to different treatment models. The traditional hospitalization time for ACLR is 7 to 11 days. During this period, patients can receive continuous psychological care, pain care and postoperative rehabilitation guidance before and after surgery,^[32]^ which makes patients feel safe and reduces the occurrence of kinesiophobia. However, due to the time constraints of the day surgery model, nurses can only provide necessary guidance to patients in a very short time. Postoperative rehabilitation and functional exercises need to be carried out by patients at home. The psychological fear of postoperative movement of patients is ignored, resulting in a higher incidence of kinesiophobia. Both inpatients and day surgery patients can start by strengthening their understanding of the disease, understanding the surgical method and postoperative rehabilitation exercise plan, so as to reduce the fear brought by the unknown. Coronado^[33]^ conducted an 8-week cognitive behavioral physical therapy intervention on 8 patients, and the results showed that the kinesiophobia scores of 7 patients decreased. Currently, there are not many intervention studies on kinesiophobia after ACLR surgery, most of which focus on relieving pain, setting individualized rehabilitation goals, and choosing a reasonable rehabilitation training intensity.^[34]^ In the future, a set of effective intervention programs for reducing kinesiophobia after ACLR surgery should be developed to alleviate patients’ kinesiophobia in multiple stages after surgery and discharge.

### 4.2. The specificity of post-ACLR kinesiophobia tools is low, and there is an urgent need to develop targeted measurement tools

TSK is one of the most widely used tools for assessing the severity of patients’ phobia at home and abroad. It originated in the United States and has been translated and used in Finland, Turkey, Spain, Switzerland, Italy and other countries.^[35]^ In the literature included in this article, Chinese scholars all used the Chinese version of TSK-17 as a measurement tool for postoperative phobia after ACLR. Although cultural adaptation has been carried out, it is still difficult to fully apply it to postoperative phobia patients after ACLR in my country. The scale was originally compiled to assess phobia in patients with chronic low back pain,^[36]^ and then gradually expanded to postoperative orthopedic patients.^[37]^ The 17 items after Chinese translation have high universality and can reflect individuals’ kinesiophobia, perception of pain and concerns about injury risks from multiple perspectives. However, it has high universality but low specificity. In order to obtain a highly targeted measurement tool for kinesiophobia in a specific field, Swedish scholars Bäck^[38]^ adapted the TSK scale for kinesiophobia in 2012 and produced the Tampa Scale for Kinesiophobia Heart (TSK-SV Heart). This scale is the 1st tool to measure the level of kinesiophobia in patients with heart disease. Similarly, the rehabilitation process of patients after ACLR surgery is unique, such as postoperative knee function recovery and concerns about returning to sports, which are different from the manifestations of kinesiophobia in patients with chronic low back pain. Therefore, a scoring scale for kinesiophobia after ACLR surgery should be developed based on the TSK scale in the future. Taking into full consideration the fear of knee joint activities, functional exercises, and returning to sports in various stages of postoperative rehabilitation in patients after ACLR surgery, a specific tool for measuring kinesiophobia after ACLR surgery should be developed.

### 4.3. Analysis of influencing factors of kinesiophobia in patients after ACLR surgery

#### 4.3.1. General factors

The occurrence of kinesiophobia in patients after ACLR surgery is affected by demographic factors such as gender, age, education, and income. The study by Rips^[21]^ pointed out that both sexes showed a high incidence of kinesiophobia after ACLR surgery, with the incidence in men being 54% and in women being 35%. In the mid-term after ACLR, the risk of kinesiophobia in men was significantly higher than that in women. This may be related to the social role pressure that men bear in society and family. Occupational and family needs make kinesiophobia more prominent in men. However, Kuenze et al found in a study of 45 men and 45 women that there was no gender difference in kinesiophobia after ACLR.^[39]^ This may be because women have a lower subjective perception threshold for pain and are more sensitive to pain than men.^[18]^ Because they pay more attention to pain perception, the difference with men is masked, so there is no gender difference. The inconsistency of the conclusions of these 2 studies may be related to the small sample size and the inconsistent tools for assessing kinesiophobia. In clinical practice, we found that older patients with low educational levels have difficulty maintaining a rational and objective attitude when facing disease treatment and rehabilitation due to their lack of knowledge about the disease, which makes them more likely to develop a fear of exercise. On the other hand, patients with higher educational levels have a better ability to obtain disease-related information and self-management skills.^[40]^ At the same time, for patients with poor economic conditions, such as the unemployed, the jobless, and low-income people, the financial pressure brought by medical expenses becomes a heavy burden, and this worry often causes them to delay the treatment and recovery process of the disease.^[41,42]^ Furthermore, these patients usually bear heavy family responsibilities, and most of their time and energy are consumed by household chores, leaving them with less leisure time for exercise and less energy to learn about the disease. The dual dilemma of lack of exercise and disease knowledge increases the possibility of them developing a fear of exercise.

#### 4.3.2. Disease characteristics and treatment factors

Several studies^[14,16,18,29]^ have shown that higher knee pain intensity is closely related to increased kinesiophobia scores. Both the surgery itself and postoperative rehabilitation exercises will cause patients to experience pain discomfort. Pain, as a strong stimulus, will significantly enhance patients’ self-protection awareness, lead to a false perception of pain, and believe that pain will threaten the body, thereby delaying or refusing exercise. At present, music therapy, virtual reality technology, multimodal analgesia and other forms can be used to meet patients’ analgesia needs and reduce the occurrence of kinesiophobia in patients after ACLR surgery. The timing of surgery after injury is also a key influencing factor. Theunissen^[29]^ pointed out that the extension of the time interval between surgery and injury significantly increased the patient’s level of kinesiophobia at 3 months after surgery. For every month of surgery delay, the TSK-17 score increased by an average of 0.85 points. This was also confirmed by the study of Rips,^[21]^ who found that among female patients, the waiting time from injury to surgery for kinesiophobia patients was significantly longer than that for patients without kinesiophobia. This may be because long-term pain and knee instability have caused severe psychological trauma to patients and increased their fear.

#### 4.3.3. Social and psychological factors

Studies have shown that pain catastrophizing is also closely related to kinesiophobia in patients after ACLR surgery. Pain catastrophizing is a psychological cognition of an individual towards pain, which is generated by the interaction of internal personality traits and self-repetitive thinking.^[14]^ Patients with low pain thresholds are highly sensitive to pain but have low tolerance. When such patients experience unbearable pain, they may exaggerate the pain, develop a strong fear and avoidance of movement, and seriously hinder the recovery process. Therefore, it is crucial for patients to correctly understand the disease and the pain caused by the treatment process. Clinical medical workers can change patients’ cognition of pain through cognitive and behavioral therapy, mindfulness meditation, acceptance and commitment therapy, etc, thereby reducing patients’ pain catastrophizing and reducing the level of kinesiophobia in patients after ACLR surgery.^[43]^

## 5. Conclusion

This paper uses the scoping review methodology as a framework to analyze the literature on the influencing factors of kinesiophobia in patients after ACLR surgery. The analysis of the literature shows that the incidence of kinesiophobia in patients after ACLR surgery is currently high, and its influencing factors mainly include general influencing factors, disease characteristics and treatment factors, and social and psychological factors. Although the factors are divided into 3 major themes, each theme is not independent, but can interact with each other. In terms of assessment tools, there are differences in the assessment tools for kinesiophobia at home and abroad, but all countries have adopted a kinesiophobia scoring scale with strong universality, and lack a highly specific scoring scale related to ACL injury or knee injury. At present, research in this field is mainly concentrated in China and the United States, and there are fewer studies in other regions. This to a certain extent reflects that other regions do not pay enough attention to the problem of kinesiophobia in patients after ACLR surgery. Most of the current studies are cross-sectional surveys, lacking targeted intervention studies and clinical verification. In the future, longitudinal studies and cohort studies can be carried out on the basis of existing studies to explore the causal relationship between the influencing factors of kinesiophobia and kinesiophobia in patients after ACLR surgery, so as to provide patients with targeted intervention measures and support, reduce the incidence of kinesiophobia in patients, and return to sports as soon as possible.

## Acknowledgments

We thank the authors of the primary studies included in this review for their contributions to the evidence base and the team who assisted with the literature search and data coordination.

## Author contributions

**Conceptualization:** Xuemei Zheng, Wenling Tian, Xiangyue Liu, Huiyun Yuan, Lin Cui.

**Investigation:** Xuemei Zheng.

**Methodology:** Xuemei Zheng, Mengjing Chang.

**Writing – original draft:** Xuemei Zheng, Xiangyue Liu.

**Writing – review & editing:** Mengjing Chang, Wenling Tian, Dongfa Liao, Lin Cui.

## Supplementary Material


