# Supplementary material for: Influencing factors of kinesiophobia in patients after anterior cruciate ligament reconstruction: A scoping review
Source: Medicine (Baltimore). 2025 Oct 10;104(41):e45138. doi: 10.1097/MD.0000000000045138 (PMC12517816; doi:10.1097/MD.0000000000045138)
Supplement: Supplementary file 1 [file medi-104-e45138-s001.pdf]

Supplementary file 1 Literature search strategy.

PUBMED

#1 "Anterior Cruciate Ligament"[Mesh]
#2 (Cruciate Ligament,Anterior) OR (Anterior Cruciate Ligaments) OR (Cruciate Ligaments, Anterior) OR (Ligament, Anterior Cruciate)OR (Ligaments,Anterior Cruciate) OR (Anterior Cranial Cruciate Ligament) OR (Cranial Cruciate Ligament) OR (Cranial CruciateLigaments) OR (Cruciate Ligament, Cranial) OR (Cruciate Ligaments, Cranial)OR(Ligament, Cranial Cruciate) OR (Ligaments, Cranial Cruciate) OR (ACL)
#3 #1 OR #2
#4 "Kinesiophobia"[Mesh]
#5 (Pain-Related Activity Avoidance) OR (Activity Avoidance, Pain-Related) OR (Avoidance, Pain-Related Activity) OR (Pain Related Activity Avoidance) OR (Movement Phobia) OR (Fear of Movement) OR(Movement Fear) OR (Kinesophobia) OR (Kinetophobia) OR (Phobia, Movement) OR (fear avoidance model)
#6 #4 OR #5
#7 #3 AND #6
